# Interplay Between MicroRNAs and Oxidative Stress in Ovarian Conditions with a Focus on Ovarian Cancer and Endometriosis

**DOI:** 10.3390/ijms20215322

**Published:** 2019-10-25

**Authors:** Josep Marí-Alexandre, Antonio Pellín Carcelén, Cristina Agababyan, Andrea Moreno-Manuel, Javier García-Oms, Silvia Calabuig-Fariñas, Juan Gilabert-Estellés

**Affiliations:** 1 Research Laboratory in Biomarkers in Reproduction, Gynaecology and Obstetrics, Fundación Hospital General Universitario de Valencia, 46014 València, Spain; dra.kristina.agababyan@gmail.com (C.A.); javiergoms@yahoo.es (J.G.-O.); juangilaeste@yahoo.es (J.G.-E.); 2 Department of Physiology, Universitat de València, 46010 València, Spain; apellincarcelen@gmail.com; 3 Comprehensive Multidisciplinary Endometriosis Unit, Consorcio Hospital General Universitario de València, 46014 València, Spain; 4 Molecular Oncology Laboratory, Fundación para la Investigación del Hospital General Universitario de València, 46014, València, Spain; andrea.morenomanuel@gmail.com (A.M.-M.); calabuix_sil@gva.es (S.C.-F.); 5 TRIAL Mixed Unit, Centro de Investigación Príncipe Felipe-Fundación para la Investigación del Hospital General Universitario de València, 46014 València, Spain; 6 Department of Pathology, Universitat de València, 46010 València, Spain; 7 Centro de Investigación Biomédica en Red en Cáncer (CIBERONC), 46014 València, Spain; 8 Department of Paediatrics, Obstetrics and Gynaecology, University of València, 46010 València, Spain

**Keywords:** oxidative stress, miRNAs, endometriosis, high-grade serous ovarian cancer, endometriosis-associated ovarian cancer, epithelial-to-mesenchymal transition, chemoresistance

## Abstract

Ovarian cancer and endometriosis are two distinct gynaecological conditions that share many biological aspects incuding proliferation, invasion of surrounding tissue, inflammation, inhibition of apoptosis, deregulation of angiogenesis and the ability to spread at a distance. miRNAs are small non-coding RNAs (19–22 nt) that act as post-transcriptional modulators of gene expression and are involved in several of the aforementioned processes. In addition, a growing body of evidence supports the contribution of oxidative stress (OS) to these gynaecological diseases: increased peritoneal OS due to the decomposition of retrograde menstruation blood facilitates both endometriotic lesion development and fallopian tube malignant transformation leading to high-grade serous ovarian cancer (HGSOC). Furthermore, as HGSOC develops, increased OS levels are associated with chemoresistance. Finally, continued bleeding within ovarian endometrioma raises OS levels and contributes to the development of endometriosis-associated ovarian cancer (EAOC). Therefore, this review aims to address the need for a better understanding of the dialogue between miRNAs and oxidative stress in the pathophysiology of ovarian conditions: endometriosis, EAOC and HGSOC.

## 1. Introduction

**Figure 1:**
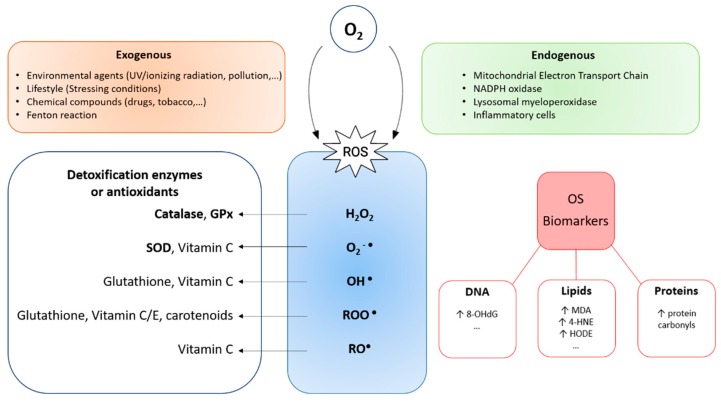
Diagram representing reactive oxygen species (ROS) production, their detoxification mechanisms and the oxidative stress alterations produced by their action, which can serve as oxidative stress (OS) biomarkers. Abbreviations: NADPH, nicotinamide adenine dinucleotide phosphate; GPx, glutathione peroxidase; SOD, superoxide dismutase; H_2_O_2_, hydrogen peroxide; O_2_^−^**^•^**, superoxide anion radical; OH**^•^**, hydroxyl radical; ROO**^•^**, p eroxyl radical; RO**^•^**, alcoxyl radical; 8-OHdG, 8-hydroxy-2′-deoxyguanosine; MDA, malondialdehyde; 4-HNE, 4-hydroxynonenal; HODE, hydroxyoctadecadienoic acid.

The Great Oxidative Event occurred between 2.4 and 2.1 billion years ago when high O_2_ concentrations appeared in the Earth atmosphere as a metabolic product of cyanobacteria oxygenic photosynthesis [1]. This phenomenon is considered a breakthrough for life on Earth, as living organisms had to develop an arsenal of antioxidant strategies to adapt to this powerful compound. However, in some circumstances the balance between oxidants and antioxidants might be shifted in favour of the former, giving raise to oxidative stress (OS). Remarkably, high partial oxygen pressures might also lead to OS through the formation of highly reactive oxygen species (ROS), including superoxide anion radical (O_2_^−^**^•^**), hydroxyl radical (OH**^•^**), hydrogen peroxide (H_2_O_2_), and peroxyl radical (ROO**^•^**) (Figure 1).

Certain levels of ROS can be produced endogenously as a sub-product of countless chemical reactions essential for cell life (including those mediated by the mitochondrial electron transport chain reactions and nicotinamide adenine dinucleotide phosphate (NADPH) oxidase), playing an important role in the regulation of cellular signalling processes [2]. However, an excess of ROS provokes the disruption of redox signalling and control and/or molecular damage [3] (Figure 1). On the other hand, ROS can also be generated exogenously, as a result of the exposure of biological systems to environmental agents (i.e., ultraviolet or ionizing radiation) or by the action of free iron (via the Fenton reaction) [4,5] (Figure 1).

The antioxidant mechanisms in charge of avoiding oxidative damage to cells include: a) enzymes (such as superoxide dismutase, glutathione peroxidase, catalase and metal binding proteins), b) non-enzymatic protectors (such as glutathione, vitamin E, vitamin C, uric acid, bilirubin and albumin) and c) repairers of damaged molecules (such as DNA repair enzymes, methionine, sulfoxide reductase) [6] (Figure 1). Contrary to the popular belief, growing evidence suggests a predominant role of antioxidant enzymes over dietary antioxidants in protection against OS [7]. When these antioxidant defences are overwhelmed (due to an excess of prooxidant substances, a deficiency of antioxidant agents or both [8]) OS produces damage to biomolecules essential for life, as lipids (malondialdehyde) [6,9], proteins (protein carbonyls) [3,9] and DNA (8-hydroxy-2′-deoxyguanosine (8-OHdG) [6,7,10] (Figure 1). This contributes to the pathophysiology of many pathological conditions such as Alzheimer’s disease [11], frailty [9], ovarian cancer [4], and endometriosis [1].

On the other hand, epigenetics refers to the heritable changes in gene function that cannot be explained by changes in the DNA sequence [12]. These changes are produced through four epigenetic mechanisms that are dynamic and reversible, and include: DNA methylation, histone modifications, chromatin remodelling, and the expression of non-coding RNAs, including miRNAs [13]. A growing body of evidence suggests that epigenetics could be involved in the pathophysiology of endometriosis [14] and that carcinogenesis cannot be explained only by DNA mutations, but also that epigenetic alterations need to be included in the equation [15]. In this respect, distinct epigenetic mechanisms could contribute to carcinogenesis either by repressing the expression of tumour suppressor genes (TSG) (i.e., DNA hypermethylation at gene promoters, over-expression of miRNA targeting TSG, histone modifications, and heterochromatin conformation at TSG coding regions) or allowing the activation of oncogenes (OG) (i.e., global DNA hypomethylation, down-regulation of miRNA targeting OG, histone modifications, and euchromatin conformation at OG coding regions).

**Figure 2:**
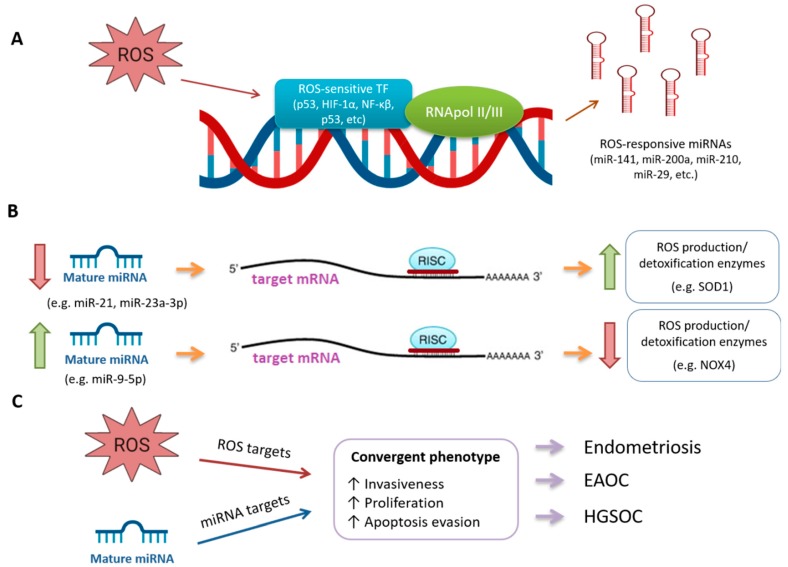
Schematic representation of the possible mechanisms of interplay between miRNAs and oxidative stress. (**A**) ROS can activate ROS-sensitive transcription factors to induce the transcription of specific primary miRNA (pri-miRNA) that will conduce to mature miRNAs; (**B**) The levels of a given miRNA inversely correlate with those of their target mRNAs, that could belong to ROS production/detoxification enzymes; (**C**) ROS and miRNAs can produce separate effects that converge in a common phenotype, leading to endometriosis, EAOC and HGOSC. Abbreviations: ROS, reactive oxygen species; TF, transcription factors; RNApol II/III, RNA polymerase II or III; HIF-1α, hypoxia-inducible factor 1α; NF-κβ, nuclear factor κβ; RISC, RNA-induced silencing complex; SOD1, superoxide dismutase 1; NOX4, NADPH oxygenase 4; EAOC, endometriosis-associated ovarian cancer; HGSOC, high-grade serous ovarian cancer.

Importantly, great research endeavours have been conducted to decipher the role of miRNAs in these pathologies. miRNAs are small (19–22 nt) non-coding RNAs that can act as post-transcriptional regulators of gene expression, reducing the expression of their target mRNAs either by inhibiting its translation or by promoting its degradation. Thus, the levels of their target mRNAs are opposed to those of their targeting miRNAs (Figure 2B). It is worth mentioning that several miRNAs can target a given mRNA and a single miRNA can target several mRNAs, increasing the complexity of the regulatory mechanism mediated by these molecules [16,17,18,19]. miRNAs are involved in pivotal biological processes including development, differentiation, apoptosis, and proliferation. Remarkably, miRNAs themselves can also act as OG or TSG, depending on their targets [20]. Extensive literature supports the role of miRNAs in the development of endometriosis (reviewed in [21,22,23,24]) as well as in endometriosis-associated ovarian cancer (EAOC) [25,26] and in high-grade serous ovarian cancer (HGSOC) [27,28,29], as hereafter described.

**Figure 3:**
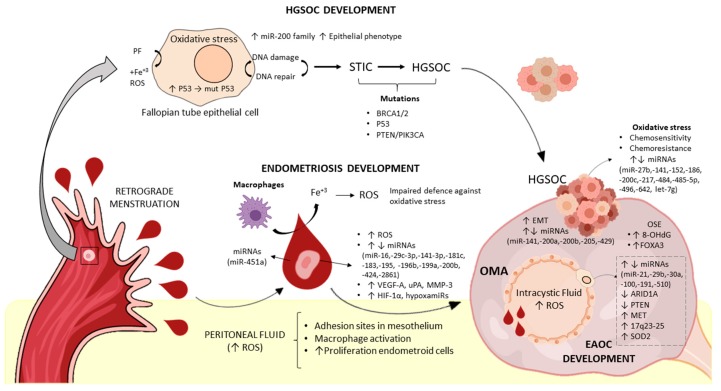
Global vision of aetiopathogenic mechanisms leading to endometriosis, EAOC and HGSOC development. Regarding HGSOC development, oxidative stress contributes to fallopian tube epithelial cells alterations, through the action of ROS. Repeated cycles of DNA damage and repair produce mutations in driver genes *BRCA1/2*, *P53*, *PTEN* and *PIK3CA*. Additionally, miRNA deregulation contributes to tumour progression. Once the malignant lesion is established in the ovary, oxidative stress is initially involved in first-line chemotherapy mechanism of action, although excessive oxidative stress is linked to tumour chemoresistance. Regarding endometriosis development, refluxed endometrial cells from patients show some features predisposing them to the development of this condition (i.e., increased angiogenesis and proteolysis, disbalanced miRNAs profile, etc). Upon menstruation, endometrial cells lose their blood supply and activate hypoxia-responsive miRNAs (hypoxamiRs) that together with erythrocyte-derived miRNAs contribute to the development of the condition. Blood decomposition by pelvic macrophages contribute to ROS production, which alters the peritoneal microenvironment to enhance endometrial cells attachment and proliferation. Finally, the intra-cystic fluid of OMAs presents with higher levels of ROS, triggering subsequent events such as miRNAs disbalance, decreased expression of ARID1A and PTEN, amplification of *MET* and 17q24–25, and increased generation of SOD2, all of which enhance the development of EAOC. Abbreviations: EAOC, endometriosis-associated ovarian cancer; HGSOC, High-Grade Serous Ovarian Cancer; STIC, Serous Tubal Intraepithelial Carcinoma; OSE, Ovarian Surface Epithelium; OMA, Ovarian endometrioma; ROS, Reactive Oxygen Species; PF, Peritoneal Fluid; BRCA, Breast Cancer gene; PTEN, Phosphatase and tensin homolog; PIK3CA, Phosphatidylinostil-4,5 Biphosphonate 3-Kinase Catalytic Subunit Alpha; VEGF-A, Vascular Endothelial Growth Factor A; uPA, Urokinase-type plasminogen Activator; MMP-3; Matrix Metallopeptidase 3; HIF-1α; Hypoxia-inducible factor 1-alpha; ARID1A, AT-Rich Interaction Domain A; MET, mesenchymal-to-epithelial transcription factor; EMT, Epithelial-Mesenchymal Transition; SOD2, Superoxide Dismutase 2; FOXA3, Hepatocyte Nuclear Factor 3-gamma; 8-OHdG, 8-Oxo-2′-deoxyguanosine.

As for the goal of this review, the complex interplay between OS and miRNAs represents an active research area in different pathologies that develops on the basis of several premises: (1) OS triggers the expression of responsive miRNAs through ROS-sensitive transcription factors (reviewed in [30,31]) (Figure 2A); (2) miRNAs can regulate the expression of enzymes involved in ROS production or detoxification (reviewed in [31,32]) (Figure 2B); (3) miRNAs and OS independently act to reach convergent phenotypes (Figure 2C).

Therefore, the aim of the present review is to compile existing evidence on how miRNAs and OS interact through the aforementioned mechanisms into the pathophysiology of three important gynaecological diseases: endometriosis, EAOC, and HGSOC.

## 2. Endometriosis

Endometriosis is an oestrogen-dependent inflammatory disorder defined by the presence of endometrial-like tissue in ectopic locations, which limits the quality of life of affected women [33,34,35]. This pathology affects 10% of reproductive-aged women from all ethnic and social groups, although the prevalence in those patients experiencing pain, infertility, or both is as high as 35%–50% [36], being the estimated prevalence of this condition around 176 million worldwide. The most frequent involvement is in the peritoneum (superficial and deep endometriotic implants) and ovaries (ovarian endometrioma (OMA) or endometriotic cysts), although cases of pulmonary [37] and cerebral endometriosis [38] have also been documented.

While a unifying theory regarding the exact aetiopathogenic mechanism of endometriosis is still lacking [13], nowadays the most widely accepted theory is Sampson’s proposal of retrograde menstruation and implantation [39]. This theory postulates that desquamated endometrial cells reach the peritoneal cavity by retrograde flow through the fallopian tubes, where they are able to implant and survive (Figure 3). Since retrograde menstruation occurs in 90% of healthy women of reproductive age with patent fallopian tubes [40], the fact that only a small percentage develops the disease suggests that there must be additional mechanisms that allow the migrated tissue to implant and survive [41].

### miRNAs and Oxidative Stress in Endometriosis

Sampson’s proposal not only postulates an origin for the endometrial-like ectopic tissue, but also provides a mechanism for the action of OS in the pathogenesis of endometriosis. Remarkably, compelling evidence demonstrates an increase in OS markers in several fluids (serum, peritoneal and follicular fluid) and tissues in women with endometriosis (reviewed in [42,43]).

From the point of view of the desquamated cells, several studies including ours have observed that eutopic endometria expresses higher levels of vascular endothelial growth factor A (VEGF-A) (the main pro-angiogenic factor) [44,45], urokinase-type plasminogen activator (uPA), and matrix metallopeptidase 3 (MMP-3) (proteolytic factors) [46] in comparison to control endometria, a process that could be mediated by miRNAs [47]. Upon menstruation, endometrial cells lose their blood supply, entering into a hypoxic state by the time they reach the peritoneal cavity. This upregulates the expression of hypoxia-inducible factor 1-alpha (HIF-1α), which further stimulates the transcription of several hypoxia-inducible miRNAs or hypoxamiRs [30,48], as the prototypical hypoxamiR, miR-210, which is overexpressed in OMA tissues, promoting cell survival [49] (Figure 2A).

Once in the peritoneal cavity, endometrial cells floating in a mixture of blood and peritoneal fluid (PF) need to undergo a continuum of important events if they are to implant and survive. These events include the attachment to ectopic sites, extracellular matrix degradation, invasion, and angiogenesis (a complex and sequential process devoted to the formation of new blood vessels from pre-existing ones, to assure oxygen and nutrient supply for proliferation and survival [50]). Notably, in vitro studies revealed that several miRNAs are involved in distinct processes leading to the establishment and survival of the endometriotic lesions, including invasiveness (miR-200b [51], miR-183 [52], miR-199a [53]), proliferation (miR-210 [54], miR-200b [51], miR-2861 [55], miR-195 [56], miR-196b [57]), apoptosis evasion (miR-181c [58], miR-141-3p [59], miR-2861 [55], miR-195 [56], miR-196b [57], miR-210 [54], and increased angiogenesis (miR-16, miR-29c-3p, miR-424 [60]) (Table 1). A myriad of studies reveals the putative role of miRNAs in endometriosis, recently reviewed by Panir and collaborators [61]. Among these miRNAs are miR-21, miR-23a-3p, and miR-9-5p, which have been linked to the regulation or redox enzymes [30] (Figure 2B).

From the point of view of the milieu into which these cells arrive, PF from patients might favour endometriosis development through several mechanisms: (a) Firstly, increased OS in PF might create adhesion sites for the migrated cells by damaging the mesothelial wall [1]; (b) acting on endometrial cells, PF from patients is involved in the over-expression of the proteolytic factors uPA and MMP-3 [62], and not yet resolved components in this biofluid increase angiogenesis by down-regulation of angiogenesis-related miRNAs (miR-16-5p, miR-29c-3p, and miR-424-5p), mainly in eutopic cells from patients, favouring their survival [60]. The putative role of OS in mediating these effects is reinforced by recent results from Wright and co-workers [63], who observed that global down-regulation in miRNAs could be recapitulated by stimulating endometrial cells with oxidized low-density lipoprotein (LDL) (an OS marker present in patient’s PF and associated with pain). These in vitro findings have also been observed in tissues, since several authors, including us, have reported an increase in VEGF-A in endometriotic tissues, which might also be regulated by miRNAs such as miR-16, miR-29c-3p, and miR-424 [45,60,64]; and (c) accompanying erythrocytes might be a source not only of OS but also of miRNAs [65], as observed by the presence of miR-451, the most abundant miRNA in erythrocytes, in PF [47]. Surprisingly, in vitro and in vivo evidence suggests that miR-451 is uptaken by endometriotic tissues, correlating its expression with survival status of the lesions [66] (Table 2, Figure 3).

Provided that several authors do not distinguish the type of ectopic lesions into their analyses, the precise effect of OS in different endometriotic lesions is difficult to evaluate. In spite of this, some conclusions can be drawn for the OMA landscape which will be commented on from the extra- to the intra-cystic space. Firstly, several authors observed an increase in 8-OHdG [79,80], forkhead box A3 (FOXA3) and advanced glycation end products [80] in the normal ovarian cortex surrounding OMAs in comparison to the normal ovarian cortex surrounding benign ovarian cysts, which might postulate OS as a specific mechanism in endometriosis. Secondly, Ngô and co-workers [81] observed that the pro-oxidant/antioxidant balance is shifted towards enhanced OS in both epithelial and stromal cells within the cyst wall, which increased cell proliferation through ERK1/2 pathway activation. Additionally, Chen and co-workers [82] observed an increase in generation of both ROS and of superoxide dismutase 2 (SOD2) by mitochondria in stromal cells, which may support the development of the disease by allowing a high metabolic rate within these lesions. Finally, the increased ROS production in endometriotic cells might also be a consequence of the pro-oxidative inner cyst fluid stimulation [83]. Interestingly, stimulation of immortalized ovarian surface epithelium (OSE) and endometrial glandular cells with endometriotic cyst content produced more ROS than treatment with non-endometriotic cyst content [84]. Altogether, the increased OS might have consequences in gene expression, rgarding the down-regulation of the tumour suppressor gene AT-rich interaction domain A (*ARID1A*) via promoter hypermethylation [85], which is considered an early event in endometriosis malignant transformation (Figure 3).

Opposite to this evidence, Santulli and collaborators [86] did not find any significant differences in protein OS markers in the PF of women with ovarian or peritoneal endometriosis when compared with control PF, in contrast to women with deep infiltrating endometriosis. This counterintuitive finding might find a rationale either when considering OMA as an encapsulated lesion within the ovary unlikely to influence PF composition, or a possible bias in these observations due to the inclusion of patients with benign pathologies in the control population.

## 3. Endometriosis-Associated Ovarian Cancer

Endometriosis malignant transformation occurs in OMA at a higher rate than in other endometriotic lesions [87], producing the so-called EAOC. This clinical entity might be the result of a sequential process of malignant transformation from endometriotic lesions through atypical endometriosis [88] and, finally, to ovarian cancer, especially to the endometrioid (EOC) and clear cell (OCCC) histological subtypes [89]. Importantly, the estimated risk of malignancy of ovarian endometriosis may be close to 1% [90,91]. This enhanced rate of transformation of OMA, together with the specific histotypes to which it leads, points to unique carcinogenic processes within these endometriotic lesions, with a plausible involvement of OS and miRNAs.

### miRNAs and Oxidative Stress in Endometriosis-Associated Ovarian Cancer

The intra-cystic fluid within OMA represents a unique milieu that may underlay the initiation of the malignant transformation process [92], probably triggered by the release of free iron as a result of monthly bleeding and the subsequent raise in OS [84] (Figure 3). At this respect, endometriotic cysts fluid present with higher levels of free iron, lactate dehydrogenase, lipid peroxide, 8-OHdG and potential antioxidant in comparison to non-endometriotic cysts and OCCC. Also, higher levels of 8-OHdG are reportedly associated with OCCC, being almost negative in EOC [93]. In agreement with these results, Fujimoto and co-workers [94] found increased levels of 8-OHdG and heme-oxygenase 1 in cyst fluid from OMA when compared to EAOC (mainly OCCC). Additionally, the expression of 8-OHdG is decreased in EAOC tissues (OCCC and EOC) when compared to paired adjacent endometriotic tissue and OMA [95]. Therefore, one might conclude that the levels of OS are higher in OMA and decrease in OCCC, and further in EOC, which suggests their involvement mainly in the initiation process of EAOC.

Regarding antioxidants, decreased MnSOD expression and increased malondialdehyde (MDA) expression has been observed in OMA and EOAC in comparison to non-EAOC and control endometria, corroborating the increased OS levels commonly found in these tissues [96]. In vitro studies denoted that the over-expression of the antioxidant lipocalin2 increases intracellular iron concentrations in OCCC cell lines but reduces the levels of ROS and DNA damage, probably through increasing glutathione, xCT (a cystine transporter protein) and CD44v (a stem cell marker), resulting in reduced apoptosis and prolonged cell survival of OCCC [97]. Altogether, it seems plausible to acknowledge that OS is involved in early steps of the malignant transformation of endometriosis.

In this context, some of the specific alterations produced by this pro-oxidant milieu have been unravelled. Opposed to HGSOC, EAOC is characterized by mutations in several genes, including the TSG *ARID1A*, *PTEN,* and the OG phosphatidylinostil-4,5 biphosphonate 3-kinase catalytic subunit alpha *(PIK3CA)* [98]. Winarto and collaborators [96] investigated tissue samples from patients with endometriosis, EAOC, or non-EAOC to observe that *ARID1A* expression decreases with increased OS a finding also corroborated in vitro. Interestingly, an epigenetic modification (*ARID1A* promoter hypermethylation in OMA) might underlay this observation [85]. In addition, chromosomal aberrations are more frequently found in EOC over OMA and less frequently in extragonadal endometriosis, which might reflect a clonal expansion of aberrant OMA cells produced as a result of the harmful intra-cystic milieu [87]. In addition, copy number variation in OCCC has been identified by several authors: for instance, mesenchymal-to-epithelial transcription factor (*MET)* gene amplification is a frequent event in OCCC and this genomic amplification can be recapitulated in an ROS-induced rat carcinoma model [99]. Furthermore, amplification at loci 17q23-25 occurs in approximately 40% of OCCC, which might over-express the encoded miR-21, decreasing the expression of their target PTEN. Therefore, both OS and miRNAs converge in some cases with genetic alterations to provoke the loss of function of important tumour suppressor genes in EAOC carcinogenesis (Figure 3).

Apart from its role in tumour initiation, a growing body of evidence has shed light into the role of deregulated miRNAs in other processes linked to EAOC carcinogenesis, as cell proliferation [100,101,102], migration [100,101], invasion [100,102], and epithelial-to-mesenchymal transition [100,103,104,105,106]. However, differences in study design and methodological approaches render low overlapping between reported deregulated miRNAs (Table 3), that is limited to miR-21 [106,107], miR-510 [104,105], miR-29b [108,109], miR-191 [102,110] and miR-30a [111,112].

Regarding treatment, first-line chemotherapy agents in ovarian cancer (EAOC and HGSOC) are represented by taxanes and carboplatin, which exert their antitumour effect partly mediated by an increase in OS (Figure 3). A very recent study from Amano and co-workers [114] observed an association between higher mitochondrial SOD2 levels in EAOC and poor prognosis, without any difference regarding histology (OCCC vs. EOC), which could reflect an improved scavenging of platinum-mediated ROS production during EAOC treatment. On the other hand, the acquisition of chemoresistance by both EAOC and HGSOC is directly linked to the poor overall results in these patients. Specifically, the clear cell subtype is known to be more chemorresistant and associated to worse prognosis than the endometrioid subtype.

In this context, Sugio and co-workers [108] observed that increased miR-29b levels correlate with progression-free survival in OCCC patients. Of note, the down-regulation of the protein Bcl2-associated athanogene 3 (BAG3) seemed to induce the miR-29b expression, which finally sensitized cells to paclitaxel. In an attempt to overwhelm chemoresistance in OCCC, new therapeutic approaches have been developed, including the use of the rapamycin analogue everolimus, for which resistances have also been documented [115]. However, Nagaraja and collaborators [113] found that induced over-expression of miR-100 increased the sensitivity to everolimus in OCCC cell lines, an effect mediated by the inhibition of mammalian target of ramapycin (mTOR) signalling (Table 3). All in all, these results might pave the way for a miRNA-based therapy to overcome chemoresistance in EAOC.

Therefore, there exists compelling evidence that miRNAs and OS cooperate in the distinct steps of EAOC carcinogenesis to accomplish the malignant transformation of endometriosis and that both of them influence therapeutic outcomes in these conditions.

## 4. High-Grade Serous Ovarian Cancer

Ovarian cancer is the fifth cause of cancer death in women and the most lethal of gynaecological malignancies [116]. Although this term designates a group of multiple malignant diseases sharing the same anatomical location and with different histological types [117], the poor prognosis data is mainly related to the most frequent (70%) histological subtype, HGSOC. Actually, HGSOC causes 70%–80% of gynaecological cancer-related deaths [118]. Regarding prognosis, more than 80% of HGSOC patients are diagnosed in advanced stages and the 5-year survival rates are below 50% [119,120]. This poor prognosis in HGSOC may be attributable to the asymptomatic nature, the lack of diagnostic methods in initial stages, and the presence of metastases at the time of diagnosis, although major determinants of HGSOC-related deaths are the frequent recurrences and the acquisition of chemoresistance by the tumour [121]. To this respect, slight changes have been produced in the medical treatment algorithm for HGSOC in the last decades, which relies on taxanes and cisplatin derivatives.

Despite its clinical importance, the exact aetiopathogenic mechanism of HGSOC remains elusive, since an unsolved debate on whether HGSOC arises from the ovary itself or from the fimbriae of the fallopian tubes still exists. Initially, the OSE was proposed as the focus for epithelial ovarian cancers, postulating the repeated ovulation and inclusion of cysts during regeneration as an initiation mechanism. The later theory of the fallopian tube epithelium (FTE) as a source of this tumour has gained growing attention [122], considering the premalignant lesion serous tubal intraepithelial carcinoma (STIC) as an early event. Accordingly, the tumour formed in the fallopian tube spreads to the ovary helped by the retrograde menstruation, where it is more capable of metastasizing [123] (Figure 3).

### miRNAs and Oxidative Stress in High-Grade Serous Ovarian Cancer

Multiple studies based on both OS and miRNAs have reinforced an FTE origin of HGSOC. Vercellini and co-workers [124] proposed the “incessant menstruation” hypothesis, which states that pelvic macrophages decompose menstrual blood in the peritoneal cavity, and the released free-iron can damage epithelial fimbriae via ROS production. Specifically, the action of ROS on the FTE provokes a continued process of DNA damage and repair, which permits the sequential acquisition of mutations, and genomic instability. This genomic instability is represented by very frequent structural and numerical aberrations in chromosomes 3, 8, 11, 17, and 21 [125] and the mutations in HGSOC-driver genes, breast cancer gene 1 and 2 *(BRCA1/2), TP53,* or *PTEN/PIK3CA* [126] (Figure 3). Additionally, the loss of function of these genes has been involved with an impaired defence against OS in these tumours [127], what determines a positive feed-back loop. Nevertheless, we cannot withstand that 17% of HGSOC patients carry germline mutations in BRCA1/2 [128].

From an epigenetic standpoint, DNA methylation [129] and miRNA [130] studies endorse the fallopian tube origin of HGSOC. For the former, Klinkebiel and co-workers [129] examined a small cohort of paired HGSOC, FTE, and OSE found that DNA methylomes are more highly conserved between HGSOC and FTE than between HGSOC and OSE. For the latter, Yang and co-workers [130] examined the expression of the miR-200 family (i.e., miR-200a, -200b, -141, and -429) and miR-205, their target genes and downstream effectors in a panel of HGSOC, STIC, FTE, and OSE tissues. As a result, the authors observed an over-expression of miR-200 family in HGSOC, STIC, and FTE and an increase of the epithelial phenotype through down-regulation of the target genes *ZEB1*, *ZEB2*, *TGFβ1,* and *TGFβ2*. These effects were not observed in OSE. Interestingly, pre-miR-200 transfection in FTE cells increased the levels of CA-125, recapitulating the high expression of this mucin in HGSOC.

Compelling literature shows evidence that epithelial-mesenchymal transition (EMT), a process by which epithelial cells lose their characteristic organization and acquire the motility of mesenchymal cells, plays a central role in HGSOC tumour progression and chemoresistance acquisition [131,132]. Notably, several miRNAs are among their master regulators. Boac and collaborators [133] serially treated four ovarian cancer cell lines (A2780CP, A2780S, IGROV1, and OVCAR5) with six cycles of cisplatin, and assessed the miRNA patterns in each of the treatment-recovery cycles. They identified five known miRNAs positively (namely miR-496, miR-485-5p, let-7g, and miR-152) or negatively (miR-27b) correlated with cisplatin chemoresistance, being the modulated pathways mainly involved in EMT regulation. Zhu and collaborators [134] demonstrated that decreased expression of miR-186 is associated with increased cisplatin resistance in HGSOC patients. Remarkably, decreased miR-186 levels up-regulate those of their target Twist1, an EMT driver, promoting the mesenchymal phenotype. Hellman and collaborators [135] analysed nine studies involving gene data sets to discover pathways associated with platinum resistance in ovarian cancer. Interestingly, despite the low degree of gene overlapping due to study design heterogeneity and technology employed, pathways related to OS (“oxidative stress”, “oxidative stress response mediated by nuclear factor (NF)-E2-related factor 2”) and to EMT (“TGFbeta signalling”, “cell migration”, “cellular movement”, and “cell-to-cell signalling”) were among the most over-represented in the studied datasets. In addition, components of the miR-17-92 cluster, which down-regulates two key TFGβ signalling molecules, and let-7 family members were also associated with platinum resistance in these analyses. Finally, Brozovic and collaborators [136] found that decreased miR-200s (miR-200a, miR200b, miR-200c, miR-429, and miR-141) expression is associated with a partial EMT phenotype in the ovarian cancer paclitaxel resistant cell lines OVCAR-3/TP and MES-OV/TP. Consistently, miR-200c and miR-141 inhibition increased the mesenchymal phenotype and the resistance to paclitaxel in non-resistant OVCAR-3 cell lines. As expected, miR-200c and miR-141 over-expression sensitized MES-OV/TP cells to paclitaxel through a mesenchymal-to-epithelial transition, and increased the levels of a set of redox enzymes, mainly reductases. It is important to mention that oxidative stress induces the expression of both miR-141 and miR-200c [137] (Table 4, Figure 2A). As can be observed, the vast majority of studies regarding the miRNA regulation of EMT have been developed in cell cultures. Although they represent a valuable source of information, established cell lines do not completely mirror the biological complexity of a tumour tissue sample. At the light of the importance of the EMT phenomenon in HGSOC patients’ prognosis, it becomes clear that there is a need for major number of studies in HGSOC tissue specimens that would increase the knowledge about the miRNA regulation of the EMT.

Apart from EMT, Vecchione and collaborators [138] analysed the miRNA expression profiles in 198 HGSOC patients and validated a signature of three miRNAs (miR-484, miR-642 and miR-217) involved in chemoresistance, of which, miR-484 was associated with angiogenesis regulation. In addition, Jeong and co-workers [139] observed that miR-136 behaves as a TSG and that miR-136 down-regulation is associated with poor overall results in HGSOC patients. Specifically, miR-136 targets Notch3, and miR-136 over-expression re-sensitized paclitaxel-resistant ovarian cancer cells and significantly reduced cell viability, proliferation, cancer stem cell spheroid formation, and angiogenesis, as well as increased apoptosis when compared with the effects of isolated paclitaxel treatment.

As aforementioned, first-line chemotherapy schemes (cis-platin derivatives and taxanes) in HGSOC exert their anticancer effect partially mediated by increased oxidative stress. Accordingly, Ayyagari and co-workers [142] showed a synergistical effect on reduced cell viability and increased apoptosis when ovarian cancer cell lines were simultaneously treated with the anti-parasitic drug bithionol and paclitaxel, and this effect was attributable to an increase in intracellular ROS production. These findings are in agreement with previous studies from the same research group considering the combination of biothionol and cis-platin [143].

On the other hand, several PARP inhibitors (namely olaparib, niraparib, and rucaparib) have been approved for the treatment of HGSOC. These drugs act by preventing the poly [ADP-ribose] polymerase (PARP)-mediated repair of DNA damage and are especially effective in *BRCA1/2* mutation carriers. In this respect, Hou and collaborators [144] observed that the anti-tumour effect of PARP is mediated by increased ROS production and that antioxidant treatment with *N*-acetylcysteine rescued the effect. Notably, several miRNAs have been associated with PARP inhibitors effectiveness. In vitro studies have linked miR-622 and miR-493-5p over-expression with platinum and PARP inhibitor resistance in *BRCA1* and *BRCA2* mutated ovarian cancer cell lines, respectively [145,146].

A growing body of evidence suggests that OS is involved in the acquisition of chemoresistance in HGSOC as the tumour develops (Figure 3). Belotte and co-workers [147] reported that chemorresistant MDAH-2774 and SKOV-3 ovarian cancer cell lines display a pro-oxidant state, with reduced expression of the antioxidant enzyme glutathione reductase and increased expression of reactive nitrogen species nitrate/nitrite and their synthetizing enzyme iNOs. One step further, Fletcher and co-workers [148] observed that first-line chemotherapy agents induce point mutations in key redox enzymes, allowing a pro-oxidant state in ovarian cancer cells and favouring chemoresistance. Specifically, the authors observed decreased levels of SOD2, cytochrome b-245 alpha chain (CYBA, a NADPH oxidase subunit) and glutathione reductase and an increase in iNOS activity, nitrate/nitrite levels and glutathione peroxidase in chemoresistant cells. As expected, the combination of SOD with chemotherapy (cisplatin or taxanes) significantly increased the sensitivity to chemotherapy. In addition, miR-216b increases cisplatin sensitivity by directly targeting PARP1 [140] whilst miR-9 increases sensitivity to cisplatin and PARP inhibitor by directly targeting BRCA1 [141].

Regarding the antioxidant response, Pei and co-workers [149] observed a significant inhibition of cell adhesion, migration, invasion, metastasis, and oxidative stress levels in SKOV-3 cells when treated with the antioxidant bisdemethoxycurcumin. In an interesting approach, Pons and collaborators [150] wondered whether the initial activation state of the antioxidant response could influence patients’ outcomes. The authors observed a significant reduction of the antioxidant enzymes glutathione reductase and catalase, as well as of the uncoupling proteins (UCP) UCP2 and UCP5 in HGSOC patients resistant to carboplatin/paclitaxel. Nevertheless, the small cohort assessed and the few markers of OS preclude further conclusions on this study.

Altogether, it seems clear that whichever the origin of HGSOC, both OS and miRNAs play a crucial role in its initiation, promotion, and progression, including chemotherapy outcomes.

## 5. Potential Role of miRNAs and Oxidative Stress in Diagnosis and Treatment of Endometriosis, EAOC and HGSOC

As expected, the opportunity that alterations in both miRNAs and OS markers confer as potential biomarkers of disease has not been overlooked by researchers. However, the small number of patients included in the majority of studies and the high variability in the assessed analytes preclude any molecule being proposed as a reliable biomarker at this point. A common finding in studies evaluating OS as biomarkers for endometriosis is an increase in either plasma or serum oxidative stress biomarkers [151,152] and reduced levels of thiols [153,154] in patients in comparison to control women. In addition, other authors evaluated urine as a source of biomarkers, observing higher concentration of metabolites related to inflammation and oxidative stress (namely *N*(1)-methyl-4-pyridone-5-carboxamide, guanidinosuccinate, creatinine, taurine, valine, and 2-hydroxyisovalerate) in patients with endometriosis [155]. In EAOC, two studies demonstrated the utility of examining OS-related molecules as prognosis biomarkers. Amano and collaborators [114] observed that increased levels of SOD2 in EAOC specimens associated with worse prognosis (overall survival and progression-free survival). Additionally, protease-activated receptor-2 (PAR-2) expression, which is up-regulated by OS, correlated with shorter survival in OCCC specimens [93]. Further immunohistochemical analyses also revealed that higher 8-OHdG levels is associated with poor differentiation, higher stage, and non-optimal surgical outcomes in epithelial ovarian cancer, including HGSOC, EOC, and OCCC specimens [156]. Importantly, these observations might have a counterpart in peripheral markers, since higher serum 8-OHdG levels were associated with poor prognosis and platinum resistance in epithelial ovarian cancers, especially in EOC patients [157]. Similar results were also obtained in stage I-II epithelial ovarian cancer studies [158].

Interestingly, miRNAs can be found as circulating miRNAs in a number of biofluids, including blood, urine, and peritoneal fluid [159]. Although far from the scope of this review, several mechanisms explain the higher stability of circulating miRNAs in these biofluids [160], which make them attractive biomarkers in a myriad of pathologies, including gynaecological conditions. A limited number of studies have explored the potential of circulating miRNAs as non-invasive biomarkers for endometriosis [67,68,69,70,71,72,73]. As a matter of fact, slight reproducibility has been found among studies and neither a single miRNA nor a combination of them has demonstrated a higher performance when compared to current diagnostic techniques. These differences in results might find a rationale in the type of blood sample analyzed (either serum or plasma), the distinct stages of endometriosis considered and the heterogeneous cohort considered as control. Cho and co-workers [67] found decreased levels of let-7b and miR-135 in serum samples from patients with endometriosis. Wang and co-workers [68] observed decreased levels of miR-9 *, miR-141 *, miR-145 * and miR-542-3p * and increased levels of miR-122 and miR-199a in sera from patients in comparison to control women. Interestingly, these results considering miR-122 and miR-199a have been recently corroborated by other authors [69]. Wang and co-workers [70] performed a deep sequencing approach and qRT-PCR validation to determine that down-regulated miR-30c-5p, miR127-3p, miR-99b-5p, miRNA-15b-5p, and miRNA-20a-5p and up-regulated miR-424-3p and miR-185-5p could be putative biomarkers of the disease. Regarding plasma samples, decreased levels of miR-17-5p, miR-20a, and miR-22 [71], decreased levels of miR-200a-3p, miR-200b-3p, miR-414-3p [72] and increased levels of miR-154-5p [73] have been proposed as biomarkers of endometriosis. Perhaps due to the scarcity of cases, only one study reports circulating miRNA profiles in EAOC patients [74]. Suryawanshi and collaborators identified three distinct miRNA signatures in plasma capable of discriminating among patients with EAOC, endometriosis and healthy individuals. Interestingly, four miRNAs distinguishing EAOC patients from healthy women (namely miR-15b, -16, -21, and -195) also discriminated cancer and control mice in a pre-clinical murine model. Finally, several recent studies have evidenced the utility of miRNAs as biomarkers in HGSOC. Kobayashi and co-workers [75] observed that miR-1290 is elevated in sera from HGSOC patients in comparison to control women, but not in ovarian cancers of other histological types, and that its expression correlates with tumour burden. Shah and co-workers [76] observed that the combination of sera miR-375 and CA-125 is a diagnostic biomarker of HGSOC. Todeschini and collaborators [77] employed two independent patient cohorts to identify miR-1246 as the best diagnostic marker in HGSOC. Finally, Kan and collaborators [78] built a predictive model including sera levels of miR-200b and miR-200c, with an area under the curve of 0.784, distinguishing HGSOC patients from control individuals (Table 2).

With respect to treatment, a number of studies have considered the antioxidant treatment in endometriosis, based on supplementation with Vitamin C and E [161], or preparations with different antioxidants [162,163]. As a result, peripheral oxidative stress markers diminished [161], and the pain symptoms were improved [162,163] albeit without any benefit on pregnancy rates [161]. In the last 10 years, a few clinical trials have evaluated the utility of oxidative-stress based treatments for HGSOC, either aiming to increase oxidative stress or employing antioxidants. Monk and co-workers report a very recent two-stage phase II clinical trial, which failed to provide any benefit in objective tumour responses in a panel of platinum-resistant recurrent ovarian, tubal and peritoneal cancer patients treated with i.v. elesclomol, a ROS inducer, plus weekly paclitaxel in comparison to paclitaxel alone [164]. This observation might be in agreement with the involvement of ROS in the acquisition of chemoresistance by the tumour. In agreement with this rationale, a recent phase II trial evaluated the combination of the vitamin E analogue, delta tocotrienol, with bevacizumab in 23 patients with refractory ovarian cancer. The combination produced an improvement in progression-free survival (median 6.9 months) and overall survival (median 10.9 months) with regards to the data in current literature [165]. Trudel and co-workers [166] performed a two-stage, single-arm, phase II study (NCT00721890) of the tea drink enriched with the polyphenol epigallocatechin gallate (EGCG) for maintenance treatment in advanced ovarian cancer. Sixteen participants (13 HGSOC and three EOC) were included in the study since they were in complete remission after completion of their first line treatment and were followed for 18 months thereafter. Unfortunately, daily nutritional intervention with 500mL of the drink failed to prove any benefit regarding recurrence improvement at 18 months.

Regarding miRNAs, the vast literature involving the outstanding role of miRNAs in the three considered gynaecological conditions pave the way for a miRNA-based therapy in order to restore deregulated miRNA levels. Indeed, pathologically down- or over-expressed levels of a given miRNA could be in vitro restored by the employment of miRNA mimics or antimiRs, respectively. Although a limited number of miRNAs has been tested in clinical trials (i.e., anti-miR-122 for Hepatitis C, antimir-103/107 for type 2 diabetes and non-alcoholic fatty liver diseases, antimir-155 for cutaneous T cell lymphoma and mycosis fungoides, miR-29 mimic for scleroderma, miR-16 mimic for mesothelioma and non-small cell lung cancer, and miR-34 mimics for multiple solid tumors (reviewed in [167]), none of them have reached phase III clinical trials and there is no single ongoing trial considering gynecological diseases, to the best of our knowledge. Several limitations might provide a rationale for the lack of translation of miRNA-based therapeutics, as the high probability of off-targets cellular and systemic effects (provided the multiple mRNAs targeted by a single miRNA and the difficulty of delivering miRNA therapies to a specific organ or even to a specific cellular type, respectively). However, a major limitation might lay in the lack of knowledge of the dynamic expression of miRNA patterns and the overall effect produced due to the interaction among them, since researchers usually only have access to a still picture of disease specimens.

## 6. Conclusions

In conclusion, extensive literature shows that there exists an interplay between miRNAs and oxidative stress in several gynaecological conditions, highlighting endometriosis, EAOC and HGSOC. In endometriosis, oxidative stress and miRNAs contribute to the establishment and development of endometriotic lesions. Regarding OMA, repeated ROS stimulation and miRNA deregulation on the cells of the cystic wall seem to play an important role in the malignant transformation of endometriosis. Besides, a mechanistic model for oxidative stress and miRNAs in OMA malignant transformation may be established: Within endometrial cysts, oxidative stress might be involved in the carcinogenesis of EAOC. Initially, repeated intra-cystic bleeding generates ROS and OS that act on the cystic wall cells. To cope with this adverse event, OMA epithelial cells increase their antioxidant response, but eventually are overwhelmed by repeated ROS, producing genetic and epigenetic alterations in crucial tumour suppressor genes. Additionally, miRNAs can contribute not only to the loss of function of these tumour suppressor genes but also to important carcinogenic events. Yet, once EAOC is established, this increased antioxidant response diminishes the platinum-mediated ROS injury and the efficiency of the chemotherapy treatment, which is also modulated by specific miRNAs. Finally, oxidative stress and miRNA deregulation is involved in the carcinogenesis of HGSOC and crucially influences the response to first-line chemotherapeutics, both regarding initial treatment outcomes and acquisition of chemoresistance.

With respect to diagnosis, increased circulating levels of OS markers have been involved with endometriosis diagnosis, and increased circulating levels of SOD and OS markers have been associated with poor prognosis in EAOC and HGSOC. Additionally, several studies have proven the putative role of miRNAs as biomarkers of these three gynaecological conditions. Regarding therapeutics, antioxidant treatment in endometriosis seems to improve the associated pain and the combination of antioxidants with bevacizumab is a promising approach in refractory ovarian cancer patients. On the other hand, miRNA treatment is still in its infancy, with few ongoing clinical trials and none of them in gynaecological diseases. The difficulty in developing miRNA-based therapies could be related to the likely off-target effects and the lack of knowledge of the precise dynamics and interactions of miRNAs throughout the disease development.

Altogether, future research endeavours are guaranteed to enlarge the knowledge on the action of miRNAs and oxidative stress in the pathophysiology of these important gynaecological pathologies and to propose targeted therapeutic strategies to deal with their pernicious effect.

## Figures and Tables

**Table 1:** Deregulated miRNAs in selected in vitro studies in endometriosis.

References	Main Biological Function Promoted	Experimental Design	Main Deregulated miRNAs in Patients
[49]	Cell survival	OMA cell line under hypoxia	↑ miR-210
[51]	Invasiveness	Immortalized endometriotic cell line 12Z, the stromal cell line ST-T1b and primary endometriotic stromal cells	↓ miR-200b
[52]		Primary eutopic and control stromal cells	↓ miR-183
[53]		Primary ectopic, eutopic and control stromal cells	↓ miR-199a
[54]	Proliferation	Primary ectopic and control stromal cells	↑ miR-210
[51]		Immortalized endometriotic cell line 12Z, the stromal cell line ST-T1b and primary endometriotic stromal cells	↓ miR-200b
[55]		Ectopic endometrial cells	↓ miR-2861
[56]		Primary ectopic, eutopic and control stromal cells	↓ miR-195
[57]		Primary ectopic and control stromal cells	↓ mi-196b
[58]	Apoptosis evasion	Endometrial cell lines	↑ miR-181c
[59]		Ectopic endometrial stromal cells	↓ miR-143-3p
[55]		Ectopic endometrial cells	↓ miR-2861
[56]		Primary ectopic, eutopic and control stromal cells	↓ miR-195
[57]		Primary ectopic and control stromal cells	↓ mi-196b
[49] [54]		OMA cell line under hypoxia Primary ectopic and control stromal cells	↑ miR-210
[60]	Angiogenesis	Primary ectopic, eutopic and control stromal cells	↓ miR-16, ↓ miR-29c-3p, ↓ miR-424

↑, up-regulated levels; ↓ down-regulated levels; OMA: ovarian endometrioma.

**Table 2:** Deregulated miRNAs in selected studies considering distinct biofluids from patients with endometriosis, EAOC or HGSOC compared to control women.

miRNAs in Biofluids			
Reference	Gynaecological Condition	Biofluid Specimen	Main Deregulated miRNAs in Patients
[47]	Endometriosis	Peritoneal fluid	↑ miR-106b-3p, miR-451a and miR-486-5p
[67]		Serum	↓ let-7b and miR-135
[68]		Serum	↓ miR-9 *, miR-141 *, miR-145 * and miR-542-3p ↑ miR-122 and miR-199a
[69]		Serum	↑ miR-122 and miR-199a
[70]		Serum	↓ miR-30c-5p, miR-127-3p, miR-99b-5p, miRNA-15b-5p and miRNA-20a-5p ↑ miR-424-3p and miR-185-5p
[71]		Plasma	↓ miR-17-5p, miR-20a and miR-22
[72]		Plasma	↓ miR-200a-3p, miR-200b-3p and miR-414-3p
[73]		Plasma	↑ miR-154-5p
[74]	EAOC	Plasma	Three distinct miRNA signatures, including ↑ miR-15b, miR-16, miR-21, and miR-195
[75]	HGSOC	Serum	↑ miR-1290
[76]		Serum	↓ miR-375 + CA-125 levels
[77]		Serum	↑ miR-1246
[78]		Serum	↑ miR-200b, miR-200c

↑, up-regulated levels; ↓ down-regulated levels; CA-125, cancer antigen 125.

**Table 3:** Deregulated miRNAs in selected studies in EAOC.

Reference	Effect	Experimental Design	Main Deregulated miRNAs in Patients
[100]	Promoted proliferation, migration, invasion	OCCC and adjacent non-tumor tissues	↓ miR-424
[101]	Increased cell motility, growth and colony formation	OCCC and EOC cell lines and OMA primary stromal cells	↓ miR-381
[102]	Increased cell proliferation and invasion	EOC, OCCC, OMA and control endometria tissues	↑ miR-191
[103]	Increased MET phenotype and good prognosis	HGSOC, EOC, OCCC and mucinous ovarian cancer tissues	↓ miR-506
[104]	Increased EMT phenotype	HGSOC and OSE tissues	↑ miR-205-5p
	EMT (miR-200s), poor PFS and OS (miR-200c -3p)	HGSOC, OCCC and OSE tissues	↑ miR-200s, miR-182-5p ↓miR-383
	Hystology differentiatiors	OCCC and HGSOC tissues	↑ miR-509-3-5p, miR-509-3p, miR-509-5p, miR-510
[105]	Poor overall survival	OCCC and HGSOC tissues	↓ miR-510, miR-129-3p
[106]	Increased EMT phenotype	OCCC and HGSOC tissues	↑ miR-9
[107]	Down-regulation of the TSG PTEN	OCCC tissues	↑ miR-21
[108]	Increased paclitaxel chemosensitivity	OCCC cell lines	↑ miR-29b
[109]	Poor prognosis	OCCC, HGSOC, mucinous ovarian cancer and control tissues	↓ miR-29b
[110]	Increased apoptosis evasion	EOC, OMA and control tissues	↑ miR-191
[111]	Hystology differentiatiors	OCCC, EOC, HGSOC and mucinous ovarian cancer	↑ miR-30a and miR-30a *
[112]	Poor overall survival in ovarian papillary serous carcinoma tissues	OCCC and ovarian papillary serous carcinoma tissues	↓ miR-30a, miR-30e and miR-505
[113]	Enhanced sensitivity to everolimus	OCCC and OSE cell lines	↓ miR-100

↑, up-regulated levels; ↓, down-regulated levels; EOC, endometrioid ovarian cancer; OCCC, ovarian clear cell carcinoma; OSE, ovarian surface epithelium; HGSOC, high-grade serous ovarian cancer; EMT, epithelial-to-mesenchymal transition; MET, mesenchymal-to-epithelial transition. PTEN, phosphatase and tensin homologue; TSG, tumour suppressor gene; PFS, progression-free survival.

**Table 4:** Deregulated miRNAs in selected studies in HGSOC.

Reference	Effect	Experimental Design	Main Deregulated miRNAs in Patients
[130]	Susceptibility to oncogenic mutations and histologic differentiation. FTE cells increase CA-125 upon pre-miR-200 transfection.	HGSOC, STIC and FTE vs. OSE	↑ miR-200a, miR-200b, miR-141 and miR-429 and miR-205
[133]	Five miRNAs associated with cisplatin resistance EMT phenotype associated with higher chemoresistance Two pathways associated with overall patient survival (TGF/WNT and Regulation of EMT)	Four ovarian cancer cell lines, public ovarian cancer dataset	Positively correlated namely miR-496, miR-485-5p, let-7g and miR-152 Negatively correlated miR-27b
[134]	EMT phenotype, cisplatin resistance and worse prognosis	HGSOC tissue and ovarian cancer cell lines (chemosensitive and chemoresistant)	↓ miR-186, ↑ miR-200 family (significantly miR-141 and miR-200a)
[135]	Platinum resistance, related to EMT and stemness	Exploratory study based on nine published gene sets associated with platinum resistance in ovarian cancer.	↓ miR-17-92 cluster, let-7 family members
[136]	Stronger EMT phenotype and paclitaxel resistance	Two ovarian cancer cell lines (sensitive and resistant to paclitaxel and carboplatin)	↓ miR-200s (miR-200a, miR200b, miR-200c, miR-429 and miR-141)
[138]	Increased chemoresistance by regulation of the VEGFB and VEGFR2 pathway	198 serous epithelial ovarian carcinomas, six epithelial ovarian carcinoma cell lines	↓ miR-484 (tumour angiogenesis), miR-642, miR-217
[139]	Poor prognosis, increased placlitaxel resistance	HGSOC tissues relative to normal control tissues. Placlitaxel resistant ovarian cell lines.	↓ miR-136
[140]	Decreased cisplatin resistance by PARP1 regulation	Cisplatin-resistant and cisplatin-sensitive ovarian cancer cell lines	↓ miR-216b
[141]	Longer progression-free survival (PFS), increased platinum sensitivity to cisplatin and PARP inhibitors by directly targeting BRCA1	Serous ovarian cancer patients and tumour xenografts	↑ miR-9

↑, up-regulated levels; ↓, down-regulated levels; BRCA1, Breast Cancer type 1 susceptibility protein; EMT, epithelial-to-mesenchymal transition; VEGFB, vascular endothelial growth factor B; VEGFR2, vascular endothelial growth factor receptor 2; FTE, Fallopian Tube Epithelial; HGSOC, high-grade serous ovarian cancer; OSE, ovarian surface epithelium; PARP1, Poly [ADP-ribose] polymerase 1; STIC, Serous Tubal Intraepithelial Carcinoma, TGF, Transforming Growth Factor; Wnt, Wingless-related integration site.
